# Acute respiratory failure caused by Penicillium Marneffei infection after Kidney Transplantation:A case report

**DOI:** 10.1016/j.rmcr.2024.102145

**Published:** 2024-11-21

**Authors:** Wenbo Qiao, Xinyue Xiang, Qionglin Deng, Huijuan Li, Yan Lin, Junqing Chu

**Affiliations:** a Department of Nursing, The First Affiliated Hospital, Zhejiang University School of Medicine, Hangzhou, China; b Department of Respiratory Medicine, The First Affiliated Hospital, Zhejiang University School of Medicine, Hangzhou, China; c Department of General Intensive Care Unit, The First Affiliated Hospital, Zhejiang University School of Medicine, Hangzhou, China

## Abstract

Penicillium Marneffei (PM) infections often present with nonspecific clinical manifestations, and severe respiratory failure after infection is particularly uncommon. This case report a patient who developed severe respiratory failure due to PM infection post-kidney transplantation. Additionally, it delves into pertinent treatment measures and nursing strategies. The patient was diagnosed with PM infection, respiratory failure, and post-kidney transplant status. The treatment measures included the drug therapy and dynamic adjustment the application of nitric oxide combined with ECMO-prone position ventilation, balancing bleeding and thrombosis risks, early enteral nutrition and adaptive progression to pulmonary rehabilitation exercises. This case is a valuable reference for critical care in similar conditions.

## 1 Introduction

Penicillium Marneffei (PM) constitutes a rare opportunistic pathogen endemic to Southeast Asia and the southern regions of China [1]. PM infections predominantly affect immunosuppressed or immunodeficient individuals, notably AIDS patients, where without timely diagnostic intervention, mortality rates can soar to 50.6 %–97 %, posing a grave threat to patient survival [2,3]. Nonetheless, PM infections after solid organ transplantations are comparatively infrequent. The nonspecific clinical presentations of PM infections often encompass skin and mucosal lesions, dyspnea, abdominal discomfort, distension, anemia, wasting, and lymphadenopathy, among other manifestations, complicating disease diagnosis and nursing care [4,5]. On March 29, 2024, our department received a patient with severe respiratory failure attributed to a Marneffei basket infection post-kidney transplantation. Following standardized therapeutic interventions and nursing care, the patient demonstrated notable recovery.

This retrospective case study elucidates our encounter with a patient afflicted by PM infection leading to respiratory distress. We impart valuable insights of the patient in intensive care unit and elucidates the pertinent treatment experiences.

## 2 Case presentation

The patient, a 35-year-old male, was admitted to the nephrology department due to “chest tightness and a two-day decline in urine output, occurring more than a decade post-kidney transplantation.” On March 29, 2024, he exhibited dyspnea, hypotension, and a resting SpO_2_ of 90 % while on mask oxygen inhalation, necessitating his transfer to the ICU for enhanced surveillance and intervention. ICU diagnoses included Marneffei infection, heart failure, and pulmonary infection following kidney transplantation. Physical examination revealed a body temperature of 36.9 °C, a heart rate of 118 beats/min, a respiratory rate of 26 breaths/min, and a blood pressure of 135/83 mmHg (1 mmHg = 0.133KPa). Blood gas analysis indicated a pH of 7.26, lactic acid of 1.1 mmol/L, K^+^ of 5.8 mmol/L, PCO_2_ of 29 mmHg, PO_2_ of 79 mmHg, SpO_2_ of 93 %, and an oxygenation index of 92 mmHg. Laboratory findings showed a white blood cell count of 17.96∗10^9/L, CRP of 38.83 mg/L, hemoglobin of 103 g/L, platelet count of 104∗ 10^9/L, B-type natriuretic peptide of 3850 pg/mL, and creatinine of 736 μmol/L.

Upon ICU admission, the patient was transitioned to high-flow nasal oxygen inhalation at a FiO_2_ of 85 % with a flow rate of 60L/min, maintaining SpO_2_ from 94 % to 96 %. 2 hours post-ICU admission, blood purification therapy was initiated. 5 hours later, the SpO_2_ declined without evident provocation, fluctuating between 85 % and 90 %. The patient underwent bedside intubation, and ventilator assistance was provided at a FiO2 of 100 %, with a pressure support of 20 mmHg, PEEP of 10 mmHg, achieving SpO_2_ of 85–95 %, and an oxygenation index below 100 mmHg.

14 hours post-ICU admission, a chest X-ray revealed extensive exudative processes indicative of pulmonary edema, suggesting that respiratory failure was likely induced by pulmonary infection and left heart failure. Despite a negative fluid balance of 2650 mL from blood purification and continuous dehydration over 12 hours during the night, oxygenation improvement remained marginal. Given the patient's history of long-term anti-rejection therapy and immunosuppression, a pulmonary opportunistic infection was suspected.

16 hours post-ICU admission, following electrical impedance tomography (EIT) assessment, Nitric Oxide (NO) inhalation therapy was administered. However, no significant oxygenation advancement was observed after 1 h, prompting the initiation of extracorporeal membrane oxygenation (ECMO) combined with prone position ventilation, which sustained SpO_2_ between 95% and 100 %.

By the 8th day in the ICU, the patient's oxygenation had markedly improved, allowing a reduction in ECMO support. A blood gas analysis conducted under the ventilator FiO_2_ of 50 % demonstrated: pH 7.45, PCO_2_ 30 mmHg, PO_2_ 110 mmHg, leading to the removal of ECMO. The tracheal tube was extracted on the tenth day, and high-flow oxygen was administered. On the 12th day, the patient was transferred to a general ward for further treatment.

## 3 Discussion

While in pressure control mode on the mechanical ventilator, the fraction of inspired oxygen (FiO_2_) was set at 100 %, with a pressure support of 20 mmHg and a positive end-expiratory pressure (PEEP) of 10 mmHg. However, pulse oximetry saturation (SpO_2_) was only maintained between 85 and 95 %, and the oxygenation index persistently remained below 100 mmHg. Nitric oxide (NO) inhalation therapy is known to enhance the function of type II alveolar epithelial cells and dilate microcirculation in patients, thereby improving their oxygenation status, as referenced in the literature [6]. Consequently, we considered NO inhalation therapy. Before initiating this treatment, an Electrical Impedance Tomography (EIT) assessment was performed. The results indicated poor ventilation distribution and retention in the patient's dependent lung zones. Following medical advice, arterial blood gas analysis was repeated 30 minutes after commencing NO treatment, with the patient's condition stabilized at 4-h intervals. Potential complications during NO therapy, including pulmonary edema, prolonged bleeding time, and methemoglobinemia, were closely monitored throughout [7]. Indices such as coagulation function, methemoglobin levels, and platelet counts were checked every 4 h. After one hour of NO inhalation, the patient showed no significant oxygenation improvement, leading to the recommendation of extracorporeal membrane oxygenation (ECMO) in conjunction with prone position ventilation.

The guideline [8] suggests that implementing prone ventilation for durations of 16 hours or more in patients with severe respiratory failure can lead to a reduction in mortality rates. Following admission, the patient was subjected to high-flow oxygen inhalation, mechanical ventilation, and NO inhalation; however, these interventions did not alleviate the patient's hypoxic condition. Consequently, after a multidisciplinary discussion, a treatment combining NO with ECMO-prone position ventilation was initiated. The prone position ventilation was carried out by the “Prone Position Ventilation Verification List” [9] developed by the department earlier, and goal-oriented deep sedation and analgesia were administered to maintain a RASS score of −4 to −5 points. Nasal feeding was suspended, and stomach contents were withdrawn and discarded to minimize the risk of aspiration during the turning process; particular care was taken to avoid injury to the brachial plexus and ulnar nerve associated with the prone positioning. A blood gas analysis conducted 30 minutes later showed: pH at 7.40, PCO_2_ at 25 mmHg, and PO_2_ at 80 mmHg. On the seventh day post-admission, chest and abdominal CT scans were required. The transportation of the ECMO patient within the hospital was executed following the “Standardized in-hospital Transport Instructions for Adult ECMO Patients” prepared by Chu [10], under the vigilant supervision of doctors, nurses, respiratory therapists, and transport personnel, ensuring a seamless transfer. The CT results indicated that the pulmonary exudation had reduced compared to previous scans, and there was a minimal amount of pleural effusion on both sides.

Currently, there are no established guidelines for diagnosing and treating post-solid organ transplant infection PM. Due to the rarity of the disease and the lack of large-scale related studies, anti-infection protocols are mostly based on the diagnosis and treatment guidelines for human immunodeficiency virus infection [11]. Guidelines recommend amphotericin B for the treatment of PM infection, but amphotericin B has adverse reactions such as bone marrow suppression and renal function impairment. Considering that this patient is in the post-renal transplantation state, voriconazole has minimal renal function impairment. The drug metabolism of voriconazole is nonlinear and varies among patients. Therefore, ensuring an effective blood concentration is crucial for the drug's efficacy. When the trough concentration is less than 1.0μg/mL, the treatment will be ineffective, and when the trough concentration exceeds 5.5μg/mL, drug toxicity increases. During nursing care, daily monitoring of voriconazole blood concentrations was conducted, with trough concentrations collected 15 minutes before the next dose after the fifth administration, aiming for a target range of 2.0–4.0μg/mL [12]. During ICU treatment, voriconazole levels fluctuated between 1.2–2.4 μg/mL. The dosage was dynamically adjusted during monitoring, and the blood concentration of voriconazole was eventually maintained at approximately 2.2μg/mL to ensure therapeutic effectiveness. On the fourth day of ICU admission, voriconazole administration was switched to oral doses of 100mg every 12 hours.

Early enteral nutrition is known to boost immunity, control inflammation, and aid in rehabilitation [13]. However, due to challenges such as the patient's stress state, the need for sedation and analgesia, prone ventilation, and digestive tract damage from PM infection, a multidisciplinary team devised an early nutrition plan tailored to this patient's needs. Research indicates that an enteral nutrition care protocol, guided by internal abdominal pressure monitoring, can ensure feeding safety for patients with elevated intra-abdominal pressure and notably enhance feeding compliance by minimizing feeding intolerance symptoms [14]. Consequently, a collaborative effort by the dietitian, ICU staff, and gastroenterologist led to the development of an abdominal pressure-centric enteral nutrition strategy. With the patient in a supine position, the gastric antrum motility index (MI) was assessed using ultrasound to measure gastric residual volume. Since ultrasound was not feasible when the patient was in a prone position, syringe aspiration was employed instead to gauge gastric residual volume [13]. The patient initiated enteral nutrition on the third day post-ICU admission. Starting with an internal abdominal pressure of 12 mmHg, the patient received a 500mL enteral nutrition suspension at an initial rate of 30mL/h. Over time, gastrointestinal nutrition was incrementally increased, and by the sixth day in the ICU, the Baplel enteral nutrition suspension volume was adjusted to 1500mL and maintained at a rate of 60mL/h, reaching the desired feeding goal. Throughout the hospitalization, ongoing internal balloon pressure monitoring coupled with continuous supraglottic aspiration was implemented to mitigate the risks of infection and aspiration. Albumin levels improved from 30.2g/L at ICU admission to 36.3g/L at discharge.

Upon the patient's admission to the ICU, the platelet count was 128∗10^9/L, exhibiting a downward trend, and by the fourth day, it had decreased to 35∗10^9/L. The patient received a 14-unit intravenous infusion of platelets and a daily subcutaneous injection of 3mg human interleukin-11, QD 12. Subsequent re-examination showed an increase in platelet count to 58∗10^9/L, which then stabilized between 65 and 85 ∗10^9/L, and at the time of transfer, it was 118∗10^9/L. During ECMO and blood purification therapy, the activated partial thromboplastin time was prolonged to 85.5 seconds, and the coagulation function of D-dimer was abnormal, reaching levels such as 3387ug/L. This abnormal coagulation function may be related to pathological changes caused by the primary disease, activation of the endogenous coagulation pathway due to contact between the ECMO and blood purification pipelines with blood, and the use of anticoagulants. Based on the patient's condition, ECMO can reduce the speed of anticoagulant administration, and for blood purification, heparin-free anticoagulation methods can be employed. Platelet counts, hemoglobin levels, activated partial thromboplastin time, and D-dimer were monitored every 4 h. The puncture site was evaluated for bleeding or hematoma every 4 h, and the catheter was not removed unless necessary to minimize bleeding risk. Pupil changes were observed hourly to prevent intracranial hemorrhage. Suction pressures were maintained between 80 mmHg and 120 mmHg, with gentle actions taken to prevent respiratory tract bleeding. During the ICU stay, no complications such as massive hemorrhage or deep vein thrombosis occurred.

Upon the patient's admission to the ICU, the platelet count was measured at 128∗10^9/L, showing a decreasing trend. By the 4th day, it had further decreased to 35∗10^9/L. Medical intervention included a 14-unit intravenous infusion of platelets and a daily subcutaneous injection of 3mg human interleukin-11 (QD12). Subsequent testing revealed an increase in platelet count to 58∗10^9/L, which then stabilized within the range of 65∗10^9/L ∼85∗10^9/L, and at the time of transfer, the platelet count was 118∗10^9/L. During the administration of ECMO and blood purification therapy, the activated partial thromboplastin time was found to be prolonged at 85.5 seconds, and an abnormal coagulation function was observed with D-dimer levels reaching as high as 3387ug/L. This abnormal coagulative state may be attributed to pathological changes induced by the primary disease, activation of the endogenous coagulation pathway due to contact between the ECMO and blood purification tubing with the blood, and the use of anticoagulants. Given the clinical picture, ECMO allowed for a reduction in the dosage of anticoagulants, and heparin-free anticoagulation methods were employed for blood purification. Platelet counts, hemoglobin levels, activated partial thromboplastin time, and D-dimer were monitored every 4 h to closely track the patient's condition. The puncture site was assessed for bleeding or hematoma formation every 4 h, and catheters were only removed when necessary to minimize the risk of bleeding. Pupil checks were performed hourly to monitor for signs of intracranial hemorrhage. Suction pressures were maintained between 80 mmHg and 120 mmHg, with care taken to ensure gentle suctioning to prevent respiratory tract bleeding. Throughout the ICU stay, complications such as massive hemorrhage or deep vein thrombosis were successfully avoided.

## 4 Conclusion

Marneffei infection after solid organ transplantation is relatively rare. Due to the long-term use of anti-rejection drugs, the body is immunosuppressed, which can rapidly develop into acute respiratory failure after co-infection. The disease progresses rapidly and hypoxemia is difficult to correct. In this case, the nurse actively combined nitric oxide inhalation and ECMO-prone position ventilation to improve the patient's oxygenation. The serum voriconazole concentration was monitored daily and the dose was dynamically adjusted to ensure the therapeutic effect. Early implementation of intra-abdominal pressure guidance combined with ultrasound to assess nutritional strategies to adjust heat supply in multidisciplinary collaboration; Closely monitoring the risk of bleeding and thrombosis and actively correcting anemia; Early implementation of dynamic adaptive lung rehabilitation exercise led by liaison nurses to promote early recovery of patients. In this case, voriconazole was fed nasally, and its blood concentration fluctuated greatly due to many influencing factors, so it was difficult to maintain it in an ideal state continuously. In the future, it is necessary to improve the nasal feeding program of special drugs such as voriconazole to ensure the therapeutic effect.

## CRediT authorship contribution statement

**Wenbo Qiao:** Writing – original draft, Methodology. **Xinyue Xiang:** Writing – original draft. **Qionglin Deng:** Data curation. **Huijuan Li:** Conceptualization. **Yan Lin:** Data curation. **Junqing Chu:** Writing – review & editing, Supervision.

## Patient consent for publication

The authors affirm that all content presented in this manuscript has received consent for publication in an online open-access forum.

## Ethical approval

The Ethics Committee of The First Affiliated Hospital, Zhejiang University School of Medicine approved the study.

## Funding

This research received no specific grant from funding agencies in the public, commercial, or not-for-profit sectors.

## Declaration of competing interest

No conflict of interest exists.
